# A Systematic Review of Risk Factors and Consequences of Nyaope Usage: The Illicit Street Drug Containing HIV Antiretrovirals

**DOI:** 10.1007/s10461-022-03791-6

**Published:** 2022-07-27

**Authors:** Karan Varshney, Samuel D. Browning, Sujit K. Debnath, Pavan Shet, Darshan Shet

**Affiliations:** 1 School of Medicine, Deakin University, 75 Pigdons St, Waurn Ponds, Geelong, VIC 3216 Australia; 2 College of Population Health, Thomas Jefferson University, Philadelphia, USA; 3 Faculty of Medicine, University of New South Wales, Sydney, Australia; 4 Department of Biosciences and Bioengineering, Indian Institute of Technology Bombay, Mumbai, India; 5 School of Pharmacy, Monash University, Melbourne, Australia

**Keywords:** Addiction, Antiretroviral drugs, Nyaope, Whoonga, HIV, South Africa

## Abstract

South Africa currently has the highest number of cases of HIV in the world. HIV antiretrovirals (ARVs) are publicly available across the country to address this crisis. However, a consequence of widely available ARVs has been the diversion of these drugs for recreational usage in a drug cocktail commonly known as “nyaope” or “whoonga,” which poses a significant public health concern. To better understand nyaope, we conducted a systematic review investigating the risks and consequences associated with its usage. Following the Preferred Reporting Items for Systematic Reviews and Meta-Analyses (PRISMA) guidelines, searches were conducted in eight different databases and screened thereafter. Articles were eligible for inclusion if they included analysis of least one nyaope user and considered either demographics, risk factors, or consequences of usage. Data extracted included study characteristics and limitations, as well as demographic factors, risk factors for usage in the general population, and consequences. Quality assessments were performed using the Joanna Briggs Institute’s tools. Searches produced a total of 228 articles and, after screening, a total of 19 articles were eligible for inclusion. There was a pooled total of 807 nyaope users, all in South Africa. Major risk factors for usage were being male, unemployed, not completing secondary education, pressure from peer groups, having HIV, prior use of cannabis, and to a lesser extent, usage of other substances such as alcohol and tobacco. While young adults tend to be at high-risk, evidence indicates that adolescents are also at-risk. Consequences of usage include high rates of infection, cortical atrophy, depression, and addiction. Addiction was shown to lead to individuals stealing from friends and family to pay for the drugs. HIV-positive nyaope users were more likely to partake in risk behaviours and tended to have high viral loads. Nyaope’s rise has been linked to many health and social issues. Considering that this may also disrupt HIV control efforts in South Africa, there is an urgent need to address the rise of nyaope.

**Supplementary Information:**

The online version contains supplementary material available at 10.1007/s10461-022-03791-6.

## Introduction

### HIV in South Africa

South Africa currently has the highest prevalence of human immunodeficiency virus (HIV) globally [1, 2]. In recent years, the country has taken significant steps to help more HIV-positive South African citizens gain access to treatment for this disease. Antiretroviral therapy (ART) has improved the lives of people living with HIV as this therapy has decreased mortality and morbidity, and increased lifespan. Adherence to daily oral medication is an important determinant for suppression and prevention of drug-resistant viral strains. However, maintaining adherence to daily drug intake remains a challenging task [3]. In 2010, the South Africa’s Department of Health implemented an effective program to provide increased access of antiretrovirals (ARVs), free of cost to HIV-infected South African citizens [4]. However, an unexpected result of the increased availability of treatment for HIV has been the emergence of recreational ARV use across the country.

### Recreational ARV Use

Understanding why ARVs are being consumed for purposes other than the treatment of HIV requires an explanation of the physiological effects of the drugs. Not all ARVs have neuropsychiatric effects. Efavirenz is one of the primary drugs used in ART, found to have psychoactive properties comparable to the hallucinatory drug lysergic acid diethylamide (LSD), including mania and psychosis [5, 6]. Efavirenz and another commonly used ARV known as ritonavir also seem to have specific euphoric effects when mixed with drugs such as methamphetamine, ecstasy, heroin, tobacco, and cannabis [7–9]. Mixtures of recreational drugs containing ARVs have also been found to contain rat poison, household cleaning supplies, milk powder, pool cleaner, and bicarbonate of soda [10–12]. Though users of this drug cocktail typically smoke the mixture, some individuals have been reported to inject the mixture [13]. This drug cocktail has most frequently been referred to as nyaope.

### Nyaope

Nyaope is a highly potent drug compared to other well-known drugs; while it frequently contains substances such as ARVs, cannabis, heroin, rat poison and detergent, it is worthwhile to denote the chemical makeup of nyaope has been shown to also vary and may change over time [14]. Though it is most commonly referred to as nyaope, in prior studies and media reports, this drug cocktail has also been referred to by a number of other names; these include whoonga [10, 15–17], kataza [11, 18], plazana [8, 19, 20], ungah [8, 19, 20], and BoMkon [8, 21, 22].

According to media reports, the mixture of nyaope had begun being used as early as the year 2000 [16]. However, there is a lack of concrete evidence to back up the claim by these reports that nyaope use had begun in 2000. Instead, the earliest available reports concretely documenting recreational nyaope use were published from 2006 and onwards [10, 17–21]. Based on the recognition that the usage of nyaope was becoming widespread across the country, in 2014 the South African government criminalized the possession and distribution of nyaope, with the selling of nyaope being potentially punishable with a prison sentence of up to 25 years [23].

### Objective

Despite concerns regarding the increased usage of nyaope over time [10, 24], the risk factors for nyaope use in the general population, and its consequences for users, are currently not well understood. Therefore, this paper will provide an overview of this relatively new phenomenon. Our objective is to provide a systematic review of the literature regarding the risk factors and consequences of using nyaope.

## Methods

### Database Searches

Our systematic review workflow followed the ‘Preferred Items for Systematic Review and Meta-Analyses’ (PRISMA) guidelines [25]. On April 3rd 2022, searches were conducted in eight databases: PubMed, Scopus, CINAHL, Global Health, Ovid Medline, PsycINFO, ScienceDirect, and SocIndex. Search terms in respective databases included the numerous ways in which nyaope has previously been referred to, which were the following: “Whoonga” OR “Nyaope” OR “Plazana” OR “Kwape” OR “Ungah” OR “Kataza” OR “BoMkon”. No restrictions were placed based on the date of publication.

### Screening Process

We removed all duplicate articles for the review process. Next, articles were screened for eligibility based on title, abstract, and keyword. After that, all remaining articles were assessed by full-text analysis to determine if they were eligible for inclusion in the review. Articles were included if they fulfilled the following criteria for inclusion: (1) available in English, (2) included at least one individual using nyaope, (3) analyzed demographics/risk factors for nyaope use or effects/consequences of its usage. There were no restrictions placed based on the country of study and no restrictions based on study design for original research studies, but articles were excluded if they were not original research (such as reviews, editorials, and commentaries).

### Data Extraction

Data on study characteristics was first extracted from the included studies. Extracted data on study characteristics included the following: location of study, study design, source of data, term(s) used for drug, total nyaope users compared to total participants, and limitations of the study. Next, data was extracted based on the characteristics of nyaope users. The following data was extracted for this purpose: total nyaope users, gender, age, risk factors for nyaope usage, and consequences of usage. We also included an additional column for other findings (if relevant) for each study. Examples of additional findings included length of nyaope use, pregnancy, age when individuals began using nyaope, and substance co-use.

### Quality Assessment

All studies that were included underwent a quality assessment using the Joanna Briggs Institute’s (JBI) critical appraisal tools [26]. The JBI tools were chosen as they offer a valid quality appraisal tool across multiple types of methodologies; this was important for our review as included studies had the following study designs: qualitative, case–control, cross-sectional, case report, and cohort. Following the approach conducted in other reviews [27, 28], the JBI tools were adapted to provide a numeric score, with qualitative studies and case–control studies each on ten-item scales, cross-sectional studies and case reports each on different eight-item scales, and cohort studies on an eleven-item scale. Similar to an approach previously taken [28], numeric scores were depicted graphically, with the scores being used to assess differences in methodological quality.

## Results

### Eligible Studies

**Fig. 1:**
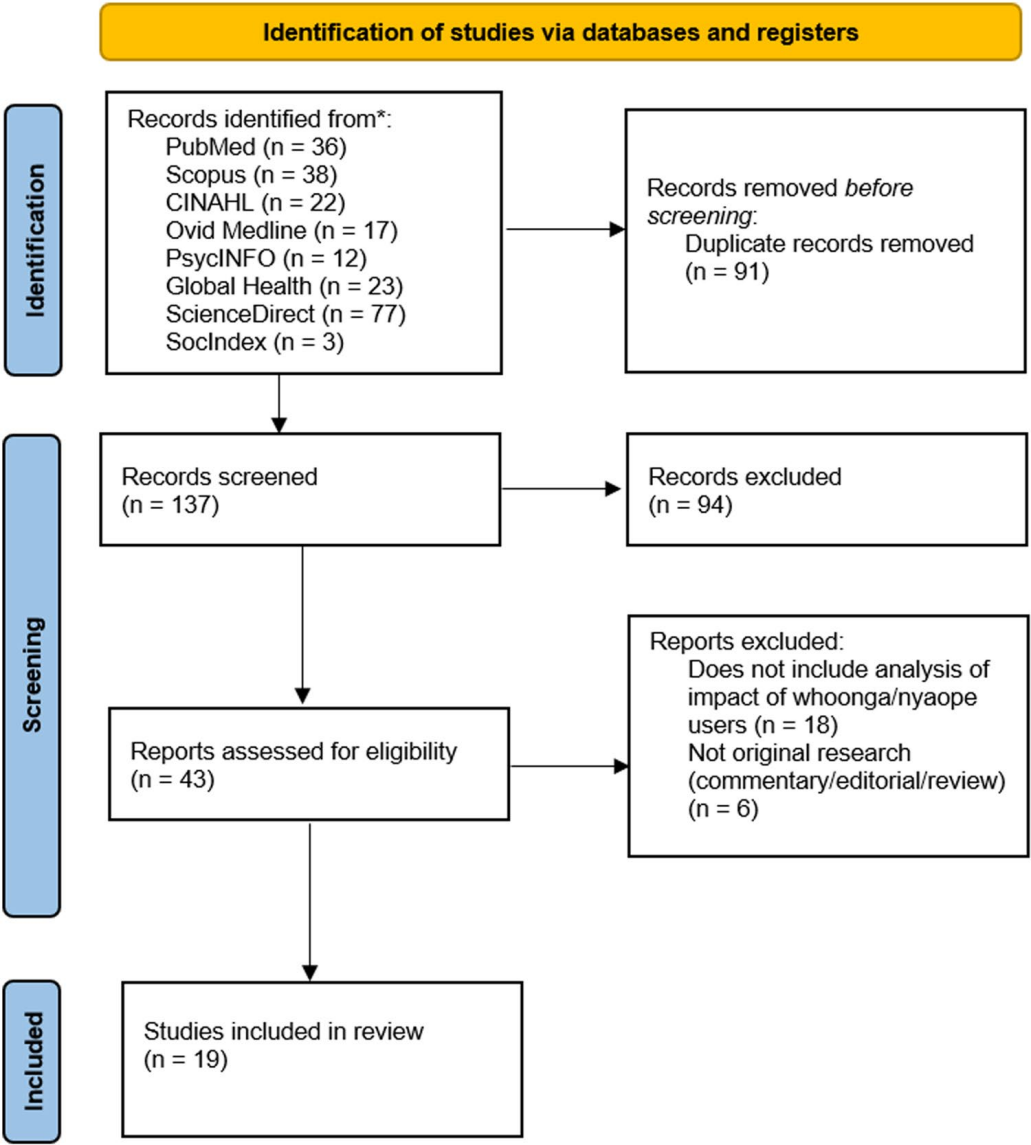
PRISMA 2020 flow diagram [25]

Combined searches from all eight databases produced a total of 228 results. 137 articles remained after the removal of duplicates. After screening by title and abstract, 43 articles remained. A total of 19 articles [6, 13, 14, 29–44] fulfilled the criteria for inclusion and were therefore eligible for analysis (Fig. 1) [25].

### Study Characteristics

All 19 studies were conducted in South Africa, with 13 taking place in Gauteng Province [13, 29–32, 35, 36, 39–44], two in Western Cape [6, 43], two in KwaZulu-Natal [33, 38], one in Mpumalanga [29], one in Northwest [29], and one in Eastern Cape [14]. One study did not specify the province within South Africa [37], whereas another study was conducted across the country [34]. An additional was study conducted in three provinces: Gauteng, Mpumalanga, and Northwest [29]. There were a range of different methodologies among included studies: six studies had a qualitative design [14, 29–33], five were cross-sectional studies [6, 13, 34–36], four were case reports [37–40], two were retrospective cohort studies [41, 42], one was a prospective cohort study [43], and one study had a case–control design [44]. The most frequently noted limitations included low participant totals in studies, study eligibility being restricted to adult participants, and a heavy reliance on self-reporting. “Nyaope” was a term used for the drug in all 19 studies, and nine studies also utilized the term “whoonga” [6, 30, 33, 35, 37, 38, 40, 41, 43]. Other terms also used were “wunga” [6, 40, 41], “pinch” [35], “unga” [35], “sugars” [37], and “kataza” [37]. The complete characteristics of the 19 included studies are listed in Table 1.

**Table 1:** Characteristics of included studies

First author [ref]	Location	Study type	Source of data	Term(s) used for drug	Total nyaope users/total participants	Limitations	Quality assessment score
DeAtley [6]	Cape Town, in Western Cape, South Africa	Cross-sectional study	Surveys	Nyaope, whoonga, wunga	6/200	Very small sample size of whoonga/nyaope users Non-representative sample of adolescents in South Africa	6/8
Fernandes [13]	Rehabilitation centers and urban areas across Pretoria, in Gauteng Province, South Africa	Cross-sectional study	Questionnaires	Nyaope	221/221	Analysis limited to a single period of time with a cross-sectional design Relying on self-reports for personal information Restricted to adult participants	4/8
Bala [14]	Mission location of Butterworth, Eastern Cape in South Africa	Qualitative study	In-depth interviews	Nyaope	7/26	Small sample size due to difficulties recruiting female adolescents Limited generalizability due to qualitative approach Inherent limitations with the usage of translators	7/10
Mokwena [29]	3 provinces in South Africa (Gauteng, Mpumalanga, Northwest Province)	Qualitative study	Focus group discussion, in-depth interviews, and participant-administered questionnaires	Nyaope	108/108	Individuals may not have disclosed factual information Lack of follow-up interviews No under-18 participants	6/10
Lefoka [30]	City of Tshwane Municipality, Gauteng Province in South Africa	Qualitative study	Semi-structured interviews	Nyaope, whoonga	24/24	Participant selection restricted by location choice and tight inclusion criteria Qualitative approach limits generalizability	9/10
Fernandes [31]	Streets from the urban areas of Ga-Rankuwa, Soshanguve and Hammanskraal of Pretoria, in Gauteng Province, South Africa	Qualitative study	Semi-structured interviews	Nyaope	68/68	Limited generalizability due to the qualitative methodology Did not consider adolescents in this study Small proportion of females, further limits generalizability	9/10
Mahlangu [32]	Hammanskraal, Gauteng Province in South Africa	Qualitative study	Semi-structured interviews	Nyaope	6/12	Small sample size Limited generalizability due to qualitative methodology	9/10
Tyree [33]	KwaZulu-Natal, South Africa	Qualitative study	Semi-structured interviews	Nyaope, whoonga	30/30	Restricted to adult patients Restricted to males Limited generalizability due to qualitative approach	7/10
Harker [34]	83 specialist treatment centers across South Africa	Cross-sectional study	Questionnaires	Nyaope	Not specified	Limited stratification by drug being used Patients who were admitted were not followed over time Not able to control for double-counting of individuals accessing substance use treatment	6/8
Moroatshehla [35]	Three townships (Mabopane, Ga-Rankuwa and Ga-Rankuwa View) of Tshwane District of Pretoria, in Gauteng Province, South Africa	Cross-sectional study	Researcher-developed demographic questionnaire, International Index of Erectile Function Questionnaire, blood samples	Nyaope, whoonga, pinch, unga	50/50	A limited sample size Total testosterone used as a proxy for bio-available testosterone Only included analysis at one point in time and therefore not longitudinal	5/8
Mokwena [36]	Substance treatment center in Tshwane, Gauteng Province in South Africa	Cross-sectional study	Self-administered questionnaire, quantitative and descriptive survey	Nyaope	141/215	Limited stratification with demographic data Cross-sectional nature of survey offers limited insights compared for realities of users compared to longitudinal design	4/8
Groenewald [37]	Peri-urban township in South Africa	Case report	Interpretative phenomenological approach, with direct accounts from participant and family member	Nyaope, whonoga, sugars, or kataza	1/1	Only a single patient Data source not specified Clinical consequences, other than addiction, not considered or explained	3/8
Mashiloane [38]	Inkosi Albert Luthuli Central Hospital, Durban in KwaZulu-Natal, South Africa	Case report	Patient data	Nyaope, whoonga	2/2	Limited to a case report for two patients Was unable to describe the extent of nyaope usage	6/8
Thomas [39]	Chris Hani Baragwanath Academic Hospital, Johannesburg in Gauteng, South Africa	Case report	Patient data	Nyaope	2/2	Limited to a case report of two patients Was unable to describe the extent of nyaope usage	6/8
Meel [40]	Chris Hani Baragwanath Academic Hospital in Soweto, Johannesburg of Gauteng Province, South Africa	Case report	Analysis of patient data	Nyaope, whoonga, wunga	3/3	Limited to a case report of only 3 patients Extent of nyaope usage not described	7/8
Meel [41]	Chris Hani Baragwanath Academic Hospital in Soweto, Gauteng Province in South Africa	Retrospective cohort study	Patient files from hospital records	Nyaope, whoonga, wunga	68/68	The retrospective design only provides limited insights about the realities and precise details of patients Single-center study, hence limited generalizability Small sample size with limited follow-up available	6/11
Dreyer [42]	Weskoppies Hospital Substance Rehabilitation Unit, Gauteng in South Africa	Retrospective cohort study	Clinical files, Substance Rehabilitation Unit (SRU) referral forms, SRU attendance, hospital computerized demographic records, nursing notes, and administration files	Nyaope	18/119	Possible issues associated with translating data, and listing of records Notable degree of missing data Limited stratification by drug of addiction	8/11
Magidson [43]	HIV voluntary testing and counselling centers in Johannesburg in Gauteng Province, and Cape Town in Western Cape, South Africa	Prospective cohort study	Surveys and assessment of viral load	Nyaope, whoonga	24/500	Lack of prior validated measures of nyaope use Was not able to determine the relationship between timing of nyaope use and viral load suppression Did not have information on viral resistance or individualized ARV regimens	7/11
Ndlovu [44]	Soweto (part of Johannesburg) in Gauteng Province, South Africa	Case–control study	MRI scans of nyaope using males and controls, along with analyses of the scans	Nyaope	28/58	Unable to account for extent of effect of confounding variables as cause of atrophy Unable to provide data explicitly linking changes in MRI to clinical symptoms	8/10

By study, the characteristics of nyaope users are listed in Table 2. There was a pooled total of 807 nyaope users. Total participants by analysis ranged from 1 to 221. Of the seven studies where both men and women were described as eligible for inclusion [13, 29, 31, 36, 41–43], six studies had a considerably higher number of male nyaope users than women [6, 13, 29, 31, 36, 41]. In one study where both men and women were eligible for inclusion, 97.1% of nyaope users were male [41].

**Table 2:** Demographics, risk factors and consequences of nyaope usage for individuals

First author [ref]	Total nyaope users	Sex (male/female)	Age	Risk factors	Consequences of usage	Other findings
DeAtley [6]	6	Not specified for whoonga users	Not specified for whoonga users	Among older adolescents, 1.22 OR of whoonga usage (95% CI 1.03–1.43; p = 0.019) For those with hazardous alcohol usage, there was a 1.80 OR for whoonga use (95% CI 1.05–3.09; p = 0.032) For those with hazardous drug use, there was a 1.62 OR for whoonga use (95% CI 1.02–2.59; p = 0.040)	N/A	Food insecurity was associated with a 0.649 OR protective effect on nyaope usage (95% CI 0.541–0.779; p = 0.000)
Fernandes [13]	221	189/32	18–23: 33% 24–29: 51.1% 30–35: 13.1% 36–41: 2.7%	90.1% did not have tertiary education 64.7% are single 54.8% began first drug use at 13–18 30.3% began first drug use at 19–24 64.3% are religious 37.5% influenced by friends to take drugs	52.0% are in rehabilitation centers 71.9% indicated that they tried stopping the usage of nyaope, but relapsed	75.5% showed an internal locus of control orientation, and 24.5% had an external orientation
Bala [14]	7	0/7	Range 15–19	3 dropped out of high school All 7 were unemployed	Could not control feelings Experienced mood swings Low motivation to study Experienced strain in relationships and impaired social functioning Regularly steal and sell from friends and family Induces hallucinations and delusions Frequent injection caused damage to the veins Unbearable stomach cramps, diarrhea, vomiting, swollen face	Users who injected tended to share needles Use of “bluetoothing”—transfusing or sharing the blood of those who were already under the influence of the drug
Mokwena [29]	108	95/13	Range 18–36 18–21: 61% 22–25: 29% 26–36: 10%	Prior substance use (52% previously used cannabis, 13% used cigarettes, 14% used other drugs) Unemployment (86%) Unfavorable social environment	27% had received rehabilitation for substance abuse Difficulty sleeping without smoking nyaope Used most of their income on nyaope Unable to control behaviour & quit Powerful addiction being difficult to control despite wanting to quit Negative view of self after beginning nyaope usage Stigma and social rejection Desire to escape their nyaope addiction	First drug used was nyaope for 21 participants Nyaope usage for 1–5 years: 38% Nyaope usage for 6–10 years: 38% Nyaope usage for 10+ years: 23%
Lefoka [30]	24	0/24	Range 22–35	HIV-positive Unemployed	Transactional sex to finance nyaope use	Nyaope is injected, increasing risk for contracting HIV
Fernandes [31]	68	61/7	Range: 18–34 18–21: 26% 22–25: 40% 26–29: 25% 30+: 9%	74% are unemployed Peer group and peer pressure increased chances of individuals experimenting with nyaope	Nyaope needed in order to be able to perform daily functions Resorting to criminal activities to be able to pay for nyaope, including stealing from family Deprioritization of hygiene Increased spread of tuberculosis Dropped out of school due to loss in concentration Regret over destruction of familial relationships Highly addictive; more addictive than other drugs used by participants in the past Increased chance of losing their job Depression, social withdrawal and anxiety, impatience, aggression	17% did not finish high school (4% with no schooling) Nyaope offers stress relief, offers confidence, and provides euphoria High availability of the drug and lack of law enforcement makes it easy to source
Mahlangu [32]	6	6/0	N/A	Limited familial support	All participants attempted treatment for addiction but relapsed, stated that the following factors contributed: lack of familial support, unprepared for treatment, lack of government support, personal issues, environmental triggers	Stated that the following would help them reintegrate into society: aftercare programs, family support after treatment, longer duration of treatment, more job and volunteering opportunities, spiritual support
Tyree [33]	30	30/0	Mean age (SD): 27 (7.0)	67% were of Black race 53% were full-time employed, 37% were unemployed 43% did not complete secondary, 83% did not complete post-secondary 97% household monthly income is > 5000 Rand 70% are Christian Nearly all participants smoked cannabis prior to beginning nyaope Peer pressure from friends Unaware of addictiveness	N/A	Overwhelming majority of users consumed by smoking
Harker [34]	N/A	N/A	The study participants were more likely to be in 25–34 compared to other age categories	Black Africans more likely than Whites Compared to those using other substances, nyaope users had a greater likelihood of being unemployed (p < 0.001), and more likely to have a secondary and tertiary education (p < 0.05) (logistic regression utilized; test values not provided)	Compared to those using other substances, nyaope users were seven times more likely to have referral for treatment by social services (p < 0.001), approximately 2.5 times more likely to be referred through the judicial system (p < 0.009) and religious groups (p < 0.001), with lower likelihoods of referrals from health service providers (p = 0.028) and schools (p = 0.001) compared to friends/family (logistic regression utilized; test values not provided)	Compared to those using other substances, nyaope users were less likely to have been tested for HIV in the past 12 months or ever (p < 0.001) (logistic regression utilized, test values not provided) Between 2012 and 2017, the range of annual nyaope-related admissions were 145–1000 (lowest in 2012, highest in 2015)
Moroatshehla [35]	50	50/0	Range 19–42 Mean (SD): 30.7 (4.8) 20–30: 27 31–40: 22 > 40: 1	Unemployment (96%) Lack of secondary education (44%) Previous use of alcohol (24%), cigarettes (12%)	Average erectile function score of 13.52 (normal = 22–25) 92% had erectile dysfunction (ED); 28% had mild ED, 20% had mild to moderate ED, 32% had moderate ED, 12% had severe ED Duration of nyaope usage positively associated (p < 0.05) with an increase in serum prolactin (95% CI 0.337–0.623; p > t = 0.00; R^2^ = 0.887) increased SHBG levels (95% CI 0.375–0.642; p > t = 0.00; R^2^ = 0.896), and decreased serum testosterone (95% CI 0.300–0.517; p > t = 0.000; R^2^ = 0.893)	Substance co-use: cigarettes (40%), cannabis (30%), cocaine (22%), alcohol (18%), mandrax (12%)
Mokwena [36]	141	Most frequently male (Exact numbers not specified for nyaope users)	Above 18 years of age (most frequently 21–30)	Prior cannabis usage Having friends who also use Pressure from friends and peers Stress Family problems Curiosity Low confidence Boredom Loneliness Unemployed	All users were admitted for treatment of nyaope addiction	Many users also utilized other substances (most frequently cannabis but also alcohol, cocaine, crystal meth, crack) Nyaope was the first substance used in 60 participants
Groenewald [37]	1	1/0	15	3 years of prior cannabis usage Peers also using nyaope	Admitted to substance abuse treatment center All money available being used for nyaope Led to criminal behaviour such as stealing from neighbours and family Failed school due to addiction	Rapid progression to daily usage Supportive mother important for patient’s rehabilitation
Mashiloane [38]	2	0/2 (Users are mothers, gender of affected infants are 1/1)	Age of mothers not specified (one infant is 7 months old, one is 9 months old)	Both mothers also abused tobacco and alcohol during pregnancy	Infants of nyaope using mother had acute malnutrition, subtle dymorphisms, intra-uterine growth restriction, autonomic instability, stridor, respiratory distress, upper airway oedema, pneumonia, septic shock, multi-organ dysfunction, prolonged hypoxia, air leak syndrome, block tracheostomy, refractory lower airway obstruction One infant died	Both mothers used nyaope while pregnant One mother was also addicted to dagga, tobacco, and alcohol
Thomas [39]	2	0/2 (Users are mothers, genders of affected infants are 1/1)	Mother ages are 26 and 29 (infant ages are 6 days and 40 days)	One mother was a tobacco smoker	One mother addicted, taking clonazepam and methadone to treat withdrawal symptoms Infants of nyaope using mothers had symmetric growth restriction, excessive sucking movements, hypoglycemia, treated with methadone, intravenous ampicillin and gentamicin, very jittery, generalized tonic–clonic seizure, audiology and retinopathy of prematurity	Both mothers used nyaope while pregnant
Meel [40]	3	3/0	29, 30 and 20	All men are HIV-positive (but none were adhering to treatment)	All patients diagnosed with infective endocarditis Two patients had right heart failure	All consumed nyaope by injection
Meel [41]	68	66/2	Mean (SD: 25.8 (4.5)	Most participants did not complete high school (mean level of completion is Grade 10) All of the participants were unemployed 76.1% were HIV-positive, the overwhelming majority of whom were not on ARV for HIV	8.2% had Hepatitis B 58.1% had Hepatitis C 36.3% were diagnosed with pulmonary tuberculosis 2.9% of patients required dialysis All patients had infective endocarditis secondary to nyaope use, with the most common clinical symptoms being dyspnoea (86.7%), fever (58.8%), right ventricular failure (42.6%), withdrawal symptoms (25.1%), and peripheral suppurative infection (8.8%) After follow up, 14.7% of patients died, 5.8% absconded, 5.8% had surgery, 4.4% had recurrent endocarditis, and 1.5% had mitral valve replacement, tricuspid valve replacement, and atrioventricular valve replacement	Median nyaope use duration (IQR): 48 (24–72) Micro-organisms responsible for infective endocarditis were *Staphylococcus aureus* (61.2%), *Enterococcus faecalis* (8.8%), *Pseudomonas aeruginosa* (1.5%), Polymicrobial infection (8.8%); 29.2% were culture negative
Dreyer [42]	18	Most frequently male (exact numbers for nyaope users not specified)	18 and above	N/A	Nyaope usage was significantly associated with non-completion of substance abuse treatment (p = 0.001; χ^2^ & Fisher’s exact test used, no exact test statistical values provided)	15% of sample used nyaope
Magidson [43]	24	11/13	Mean: 38.91 (9.24)	66.7% were HIV-positive 50% are living with depression (severe/moderate)	N/A	Recreational ARV use was not significantly associated with viral suppression at 9 months (aOR 0.58, 95% CI 0.19–1.78, p = 0.41) Majority first reported recreational ART use at 3–6 months follow-up after having initiated ART
Ndlovu [44]	28	28/0	Range 21–34 Mean (SD): 26.75 (3.79)	26 participants also reported regular alcohol and nicotine usage 28 participants utilized opioids 26 participants utilized cannabis	15 presented with symptoms of current depressive episode 12 presented with signs of antisocial behaviour Significant (p < 0.05) cortical atrophy seen in the following brain regions after FDR correction: medial orbitofrontal (p = 0.001; Cohen’s d_s_ effect size = − 0.91), rostral middle frontal (p = 0.002; Cohen’s d_s_ effect size = − 0.84), superior frontal (p = 0.0002; Cohen’s d_s_ effect size = − 1.06), superior temporal (p = 0.0005; Cohen’s d_s_ effect size = − 0.97), supramarginal (p = 0.002; Cohen’s d_s_ effect size = − 0.88)	Mean age of first nyaope usage (SD): 20.21 (3.78) Mean duration of nyaope usage (SD): 6.54 (2.97)

### Quality Assessments

**Fig. 2:**
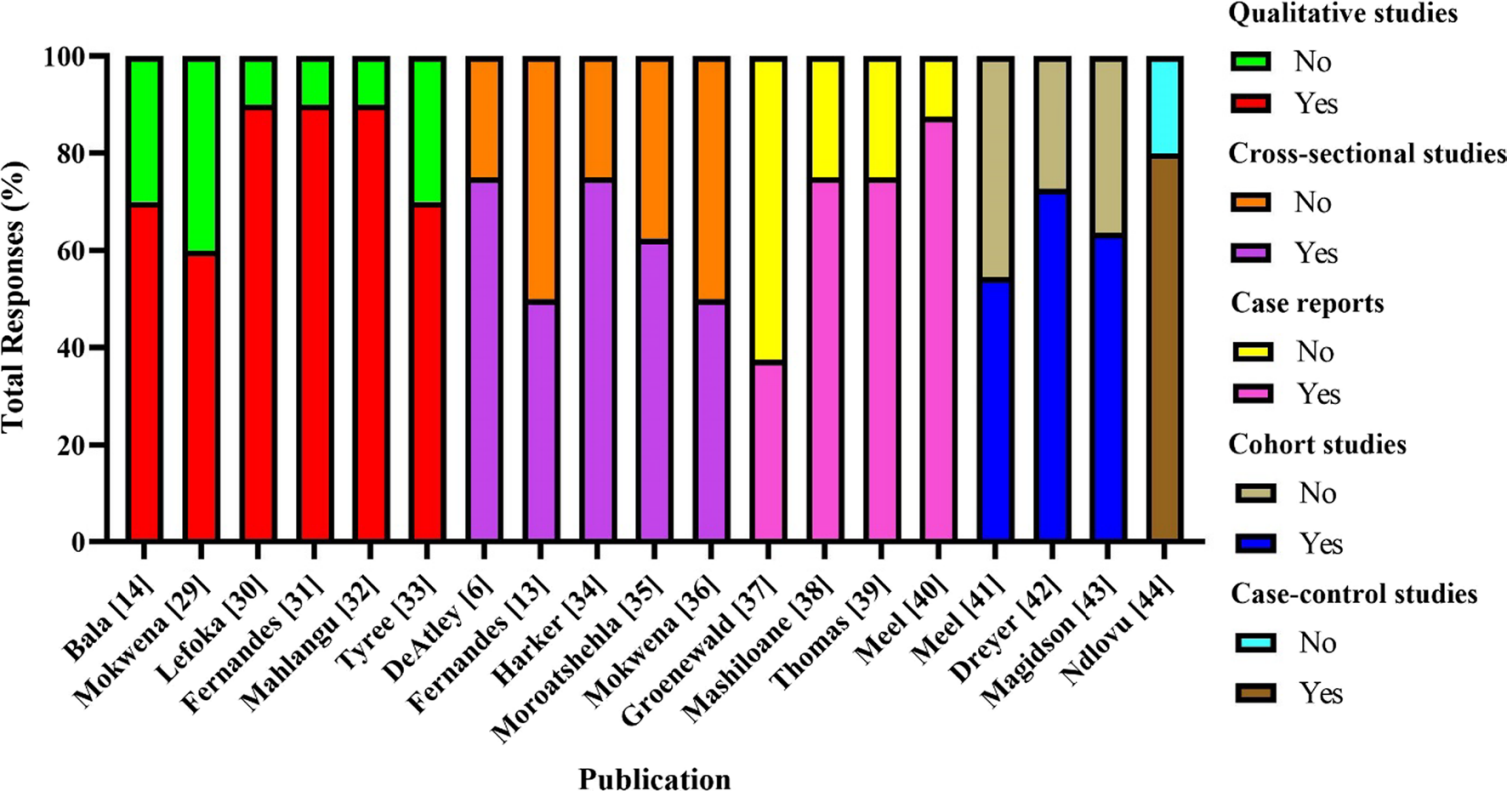
Quality assessment scores by study (Adapted from Adalbert et al. [28])

Quality assessments for all included studies are depicted in Fig. 2, and critical appraisal checklists are shown in Supplementary Tables 1–5. Studies generally ranged from mid to low quality overall. Qualitative studies had a mean score of 7.83 (SD = 1.33) on a ten-item scale, cross-sectional studies had a mean score of 5.00 (SD = 1.00) on an eight-item scale, case reports had a mean score of 5.50 (SD = 1.73) on an eight-item scale, cohort studies had a mean score of 7.00 (SD = 1.00) on an eleven-item scale, and the single case–control study had a score of 8.00 on a ten-item scale. The most frequent methodological flaws shown across studies were the limited strategies to address confounding factors, infrequent determination of the extent of nyaope use, and an inconsistent consideration of unanticipated adverse effects of usage.

### Risk Factors

In the majority of studies, nyaope users were most frequently between 18 and 29 years of age [13, 29–31, 33–36, 39–41, 44], though a number of studies also demonstrated that there are nyaope users in their 30s and 40s [13, 29–31, 34, 35, 43, 44]. While those under 18 years were not eligible for inclusion in many of the included studies, some studies showed that nyaope usage is also occurring in adolescents as young as 13 years of age [6, 14, 37].

Across the included studies, one of the most frequent risk factors for nyaope usage was unemployment [14, 29–31, 33–36, 41]. In one study, 100% of nyaope users were unemployed [41]. Other studies also had high rates of unemployment for nyaope users, at 96% [35], 86% [29], and 74% [31].

Prior substance use and co-substance use were also frequently noted as risk factors for nyaope usage [6, 29, 33, 35–39, 44]. The most commonly used substance associated with nyaope use was cannabis [29, 33, 35–37], and other substances commonly used were tobacco and alcohol [6, 29, 35, 36, 38, 39, 44]. One study found that 52% of nyaope users previously used cannabis, 13% used cigarettes, and 14% used other drugs [29]. Another study demonstrated that 92.9% of nyaope users were concurrently using alcohol and nicotine regularly and that 92.9% were concurrently using cannabis [44]. The same study found that 100% of participants were using opioids [44].

In particular, limited education and a lack of secondary education completion were also noted in several studies as common risk factors for nyaope use [13, 31, 33, 35, 41]. Other risk factors discussed were pressure to use from peers [13, 31, 33, 36, 37], being HIV-positive [30, 34, 40, 41, 43], limited familial support [32, 36], and having a Black racial background [33, 34].

### Consequences

The most commonly noted consequence of nyaope usage across studies was the intense addiction that the drug cocktail causes. Many users were receiving ongoing treatment to address addiction to nyaope [13, 29, 32, 36, 37, 42]. One study noted that nyaope use was significantly associated with non-completion of substance abuse treatment [42].

Symptoms of withdrawal, including pain [39] and an inability to sleep [29], were also described. Due to the addictive nature of nyaope, study participants described using all of their income to obtain more of the substance [14, 29, 31, 37], drop out of school [14, 31, 37], and steal from others [14, 31, 37]. These forms of theft were also reported to have additional consequences, including stigma, social rejection, and loss of family trust [14, 29, 31, 37]. One study also described that transactional sex was used to finance nyaope addiction [30].

Nyaope users had several medical complications with a wide array of clinical manifestations. Studies demonstrated nyaope users were shown to have diarrhea, facial swelling, vomiting, stomach cramps, erectile dysfunction, vein damage, right heart failure, and cortical atrophy [14, 35, 40, 41, 44]. Nyaope users were shown to be at risk for hepatitis B, hepatitis C, tuberculosis, and infective endocarditis [31, 40, 41]. Those using nyaope also showed a number of psychological symptoms including loss of behavioral control/antisocial behavior [13, 14, 29, 31, 44], negative self-perceptions [29, 36], depression [31, 43, 44], decreased motivation [14, 31], mood swings [14, 31], and hallucinations [14]. Among those with HIV, infection rates were markedly elevated [41]. It is also worth denoting that HIV-positive nyaope users were shown to be participating in transactional sex to finance nyaope use [30], were injecting the drug cocktail [30, 40], and not adhering to ARV treatment [41].

Notably, two studies included nyaope users who used the drug cocktail while pregnant [38, 39]. Of these pregnant nyaope users, one infant died, with the cause of death linked to the mother’s nyaope usage [38]. Other infants born of nyaope-using mothers had serious clinical symptoms such as septic shock, respiratory distress, seizure, multi-organ dysfunction, and retinopathy of prematurity [38, 39].

## Discussion

### Nyaope Addiction

The overall findings of this review contribute to the existing literature regarding nyaope. While it has previously been demonstrated that the emergence of this drug cocktail is a rising social issue, the risk factors for nyaope usage have not previously been understood. Furthermore, the biopsychosocial consequences of usage have not previously been described in great detail. Our findings therefore add to the existing literature in numerous ways. Our review demonstrated that being male, a teenager/young adult, unemployed, HIV-positive, and having a prior history of substance use are all major risk factors for nyaope usage. The majority of the studies included in our review were conducted in Gauteng Province. Consequences of nyaope usage have been shown to be widespread for users. These consequences include erectile dysfunction, cortical atrophy, infection, depression, mood swings, and hallucinations. Additionally, nyaope usage among pregnant mothers has been shown to be particularly dangerous. Our findings also highlight that numerous individual and societal issues arise due to the increased use of nyaope. One of the primary concerns is the highly addictive aspects of this substance, which makes it very difficult for the individual to stop using the drugs.

A study by Möller et al. provides insight into the possible neurological basis for the addictive aspects of ARVs and nyaope [9]. This study was conducted on rats and demonstrated the addictive nature of efavirenz, with numerous similarities to the psychoactive properties of methamphetamines and tetrahydrocannabinol [9]. Additionally, the seriousness of withdrawal after one ceases nyaope usage provides further insight into these drugs' addictiveness. The withdrawal effects of nyaope, which can occur for as long as about a week, include the appearance of flu-like symptoms, nausea, severe cramps, cold chills, frequent sweating, and constant diarrhea [13, 45]. When an addicted individual attempts to stop abusing these drugs, this can lead to criminality, dropping out of school, and lying to family members to facilitate continued drug use. Assisting individuals in getting through these powerful withdrawal symptoms is important for successful rehabilitation. Rehabilitation programs in South Africa can help individuals addicted to these drugs deal with withdrawal symptoms by creating more detoxification services that are unique to these particular substances [23]. It is critical to denote that while there are severe withdrawal symptoms for nyaope users, these symptoms have not been shown to occur among HIV-positive individuals who discontinue usage of their prescribed ARVs, or who miss doses of the ARVs [46]. There is hence a clear need to better understand the biological basis of withdrawal for nyaope in order to better guide the development of treatment for the withdrawal symptoms.

### Rehabilitation Programs

While addressing withdrawal symptoms is of high priority in rehabilitation programs, this alone is not adequate in fully supporting nyaope users. Several health complications can arise due to the frequent use of nyaope. Addressing these psychological and physical symptoms would be of high value in rehabilitation programs. This technique could effectively occur by including social workers, counselors, and group therapy during rehabilitation [23, 47]. Mahlangu and Geyer highlight how nyaope users expressed a desire for psychotherapy before and after their treatments [32]. Accordingly, the inclusion of such services could be of value in ensuring that individuals can work towards dealing with the psychological issues involved in this addiction. Notably, a number of nyaope users were concurrently utilizing other substances, such as cannabis, alcohol, tobacco, and methamphetamines. Rehabilitation programs therefore should also utilize approaches that are equipped to deal with the polydrug use. This will require multidisciplinary approaches that should emphasize harm reduction approaches when necessary.

To ensure that nyaope users do not eventually relapse, rehabilitation services should make efforts to ensure that former users can reintegrate into society. This also requires an understanding of what factors may have caused the individual to become addicted in the first place. Strain theories predict that impoverished individuals may resort to substance misuse as a form of “retreatism” [48]. Considering that most nyaope users are unemployed and living in poverty [23], this seems to describe the current situation in South Africa appropriately. Helping individuals find employment can hence be a valuable means of ensuring complete rehabilitation. This would involve assisting nyaope users to develop employable skills and providing them with various forms of temporary employment [45]. As unemployment is denoted as one of the most common risk factors for commencing nyaope usage, there is a need to establish efforts to increase employment levels across the nation of South Africa. This will be particularly important for unemployed, young, Black South African men, who are at the highest risk of nyaope usage.

There is a clear need for community level support to be provided by rehabilitation programs funded primarily by the public sector. With nyaope costing as low as 25 rands for a single joint, it is highly accessible in many impoverished communities [29]. However, many of the worst affected communities do not have access to affordable rehabilitation services due to limited investment for these programs in the public sector, and due to the unaffordability of many of the programs in the private sector [29]. Therefore, supportive rehabilitation programs need to be available at minimal cost for users and must be widely available in impoverished communities.

### Nyaope Prevention

Our review indicates that addressing issues of peer pressure and general ignorance of the consequences of nyaope use will be crucial for prevention. One way that this can occur is by having educators share media reports that provide truthful information about the most devastating effects of mixtures with ARVs. There is some evidence indicating that such efforts may be successful. One study demonstrated that the reason why nyaope was not popular at an educational institution was related to the prevalence of negative stigma associated with usage based on a belief that the drug composition included several dangerous substances such as painkillers, benzene, rat pellets, and ARVs [49, 50]. Accordingly, sharing media reports that highlight the worst aspects of this mixture may deter more youth in the future. It is important to denote that removal of stigma towards nyaope users in an empathizing manner will also be integral in ensuring that more users are able to feel safe and comfortable in seeking treatment. Understanding nyaope stigma can hence have a valuable role in both ensuring prevention of high-risk groups, and in increasing rates of rehabilitation for users.

A lack of public knowledge about the dangers of nyaope demonstrates that resources should be invested in informing adolescents, young adults, and the wider community about the risks of consuming this drug mixture. This can occur by creating government-funded workshops that educate individuals about the dangers of nyaope, recreational ARV use, and the dangers of addiction in general. This should also involve public broadcasts and education campaigns warning of the dangers of nyaope use. Creating educational warnings to entire communities and at-risk groups can result in lasting change that deters individuals from using nyaope [51].

Undoubtedly, addressing high levels of nyaope usage will also involve managing other forms of substance misuse among adolescents and young adults as prior substance use is a notable risk factor for nyaope usage. Younger individuals with a high level of usage of substances such as cannabis, tobacco, and alcohol tend to deal with neglect and come from challenging home environments or reside in single-parent households [18]. Reaching out to these at-risk individuals to lower levels of other substance use can potentially lead to less nyaope usage over time.

Nyaope prevention among HIV-positive individuals will be particularly important for a number of reasons. The diversion of ARV treatment can have negative consequences for the person who is no longer on treatment as they will likely have an increased viral load. This therefore increases their risk of progression to AIDS. As demonstrated in a prior study, there is also a clear need to further understand if the diversion of ARVs for nyaope is contributing to drug-resistance of treatment [52]. Considering that HIV-positive nyaope users have been shown to partake in certain behaviours which increase the risk of transmitting HIV to others, such as transactional sex and injecting with shared needles [16], there is a clear need to increase support services to HIV-positive individuals who are at risk of nyaope use. Protective and harm-reduction services, in the form of condom promotion and syringe exchange, will also offer utility in supporting this vulnerable population. Critically, there is a need to monitor the risk of violence towards HIV-positive patients and HIV healthcare providers, as both of these groups have been the victims of such violence in the past by those seeking to obtain ARVs for recreational usage [29, 53, 54]. The issues of theft and redirection of ARVs due to nyaope indicate that this is a concern for wider South African society.

### Limitations

It is important to consider and recognize the limitations of this systematic review. While several different study designs and methodologies were included, a high proportion of studies either did not include quantitative data or had a small sample size. As demonstrated by the quality appraisals, many studies were hence unable to consider confounding variables, and many adverse consequences of nyaope usage were not able to be accounted for. This indicates that a large proportion of the complex consequences of nyaope are likely to remain only partially understood. More research is thus needed on the biopsychosocial consequences of nyaope use. Furthermore, while many studies indicated that nyaope usage might be a serious issue among adolescent populations, those under 18 were not eligible for inclusion in many studies reviewed. Therefore, there is a need for larger quantitative studies that include adolescent populations. Lastly, numerous studies could not determine the temporality of factors under study. For example, it is not well established if nyaope users tend to deal with depression due to nyaope usage or if individuals dealing with depression tend to be more likely to use nyaope. Regardless of these limitations, our findings provide a useful starting point for understanding how to support nyaope users during their addiction and how to focus on at-risk populations to prevent them from starting nyaope usage.

## Conclusion

The diversion of ARVs for recreational use in the drug cocktail known as “nyaope” poses important implications for its users and their broader community. Our findings highlight that important risk factors for the use of nyaope include unemployment, non-completion of secondary school, being HIV-positive, and prior substance use. Furthermore, those of male gender and younger age are at increased risk of nyaope use; other social factors including peer pressure and stressful home environments can also lend themselves to substance use. Important medical consequences associated with the consumption of nyaope include infection risk, psychological distress, and strong addiction with severe withdrawal symptoms following cessation. Notably, the implications of nyaope use extend beyond the individual medical and psychological consequences to the wider community. Importantly, nyaope addiction can cause financial hardships and damage to family relationships, leading to a tendency for criminality and social stigmatization. In consideration of the severity of issues associated with nyaope, public health campaigns should address these documented risk factors to prevent nyaope use and reduce the burden of concomitant biopsychosocial consequences.

## Supplementary Information

Below is the link to the electronic supplementary material.

Supplementary file1 (DOCX 28 KB)

## Data Availability

The authors have provided all relevant data in our submission.
