# Supplementary material for: A Systematic Review of Risk Factors and Consequences of Nyaope Usage: The Illicit Street Drug Containing HIV Antiretrovirals
Source: AIDS Behav. 2022 Jul 27;27(2):558–77. doi: 10.1007/s10461-022-03791-6 (PMC9908705; doi:10.1007/s10461-022-03791-6)
Supplement: Supplementary file 1 — Supplementary file1 (DOCX 28 KB) [file 10461_2022_3791_MOESM1_ESM.docx]

**Supplementary Table 1: Joanna Briggs Quality Assessment for qualitative studies included in the review**

| First Author (Ref) | 1 | 2 | 3 | 4 | 5 | 6 | 7 | 8 | 9 | 10 | Total Score |
| --- | --- | --- | --- | --- | --- | --- | --- | --- | --- | --- | --- |
| **Bala [14]** | Yes | Yes | Yes | No | Yes | No | No | Yes | Yes | Yes | 7/10 |
| **Mokwena [23]** | No | Yes | Yes | Yes | No | No | No | Yes | Yes | Yes | 6/10 |
| **Lefoka [35]** | Yes | Yes | Yes | Yes | Yes | No | Yes | Yes | Yes | Yes | 9/10 |
| **Fernandes [36]** | No | Yes | Yes | Yes | Yes | Yes | Yes | Yes | Yes | Yes | 9/10 |
| **Mahlangu [37]** | Yes | Yes | Yes | Yes | Yes | Yes | Yes | Yes | Yes | No | 9/10 |
| **Tyree [38]** | No | Yes | Yes | Yes | Yes | No | No | Yes | Yes | Yes | 7/10 |

1. Is there congruity between the stated philosophical perspective and the research methodology?
2. Is there congruity between the research methodology and the research question or objectives?
3. Is there congruity between the research methodology and the methods used to collect data?
4. Is there congruity between the research methodology and the representation and analysis of data?
5. Is there congruity between the research methodology and the interpretation of results?
6. Is there a statement locating the researcher culturally or theoretically?
7. Is the influence of the researcher on the research, and vice- versa, addressed?
8. Are participants, and their voices, adequately represented?
9. Is the research ethical according to current criteria or, for recent studies, and is there evidence of ethical approval by an

appropriate body?

1. Do the conclusions drawn in the research report flow from the analysis, or interpretation, of the data?

**Supplementary Table 2.** **Joanna Briggs Quality Assessment for analytical cross-sectional studies included in the review**

| First Author (Ref) | 1 | 2 | 3 | 4 | 5 | 6 | 7 | 8 | Total score |
| --- | --- | --- | --- | --- | --- | --- | --- | --- | --- |
| **DeAtley [6]** | Yes | Yes | Yes | Yes | No | No | Yes | Yes | 6/8 |
| **Fernandes [13]** | Yes | Yes | No | Yes | No | No | Yes | No | 4/8 |
| **Harker [39]** | Yes | Yes | No | Yes | Yes | No | Yes | Yes | 6/8 |
| **Moroatshehla [40]** | Yes | Yes | No | Yes | No | No | Yes | Yes | 5/8 |
| **Mokwena [41]** | Yes | Yes | Yes | Yes | No | No | No | No | 4/8 |

1. Were the criteria for inclusion in the sample clearly defined?
2. Were the study subjects and the setting described in detail?
3. Was the exposure measured in a valid and reliable way?
4. Were objective, standard criteria used for measurement of the condition?
5. Were confounding factors identified?
6. Were strategies to deal with confounding factors stated?
7. Were the outcomes measured in a valid and reliable way?
8. Was appropriate statistical analysis used?

**Supplementary Table 3:** **Joanna Briggs Quality Assessment for case reports included in the review**

| First Author (Ref) | 1 | 2 | 3 | 4 | 5 | 6 | 7 | 8 | Total score |
| --- | --- | --- | --- | --- | --- | --- | --- | --- | --- |
| **Groenewald [42]** | Yes | Yes | No | No | No | No | No | Yes | 3/8 |
| **Mashiloane [43]** | Yes | Yes | Yes | No | Yes | Yes | No | Yes | 6/8 |
| **Thomas [44]** | Yes | Yes | Yes | No | Yes | Yes | No | Yes | 6/8 |
| **Meel [45]** | Yes | No | Yes | Yes | Yes | Yes | No | Yes | 7/8 |

1. Were patient’s demographic characteristics clearly described?
2. Was the patient’s history clearly described and presented as a timeline?
3. Was the current clinical condition of the patient on presentation clearly described?
4. Were diagnostic tests or assessment methods and the results clearly described?
5. Was the intervention(s) or treatment procedure(s) clearly described?
6. Was the post-intervention clinical condition clearly described?
7. Were adverse events (harms) or unanticipated events identified and described?
8. Does the case report provide takeaway lessons?

**Supplementary Table 4. Joanna Briggs Quality Assessment for cohort studies included in the review**

| First Author (Ref) | 1 | 2 | 3 | 4 | 5 | 6 | 7 | 8 | 9 | 10 | 11 | Total score |  |
| --- | --- | --- | --- | --- | --- | --- | --- | --- | --- | --- | --- | --- | --- |
| **Meel [46]** | No | Yes | No | No | No | Yes | Yes | Yes | Yes | No | Yes | 6/11 |  |
| **Dreyer [47]** | Yes | Yes | No | Yes | No | Yes | Yes | Yes | Yes | Yes | No | 8/11 |  |
| **Magidson [48]** | Yes | Yes | Yes | No | No | No | Yes | Yes | Yes | No | Yes | 7/11 |  |

1. Were the two groups similar and recruited from the same population?
2. Were the exposures measured similarly to assign people to both exposed and unexposed groups?
3. Was the exposure measured in a valid and reliable way?
4. Were confounding factors identified?
5. Were strategies to deal with confounding factors stated?
6. Were the groups/participants free of the outcome at the start of the study (or at the moment of exposure)?
7. Were the outcomes measured in a valid and reliable way?
8. Was the follow up time reported and sufficient to be long enough for outcomes to occur?
9. Was follow up complete, and if not, were the reasons to loss to follow up described and explored?
10. Were strategies to address incomplete follow up utilized?
11. Was appropriate statistical analysis used?

**Supplementary Table 5. Joanna Briggs Quality Assessment for case-control studies included in the review**

| First Author (Ref) | 1 | 2 | 3 | 4 | 5 | 6 | 7 | 8 | 9 | 10 | Total Score |
| --- | --- | --- | --- | --- | --- | --- | --- | --- | --- | --- | --- |
| **Ndlovu [49]** | Yes | Yes | Yes | Yes | Yes | No | No | Yes | Yes | Yes | 8/10 |

1. Were the groups comparable other than the presence of disease in cases or the absence of disease in controls?
2. Were cases and controls matched appropriately?
3. Were the same criteria used for identification of cases and controls?
4. Was exposure measured in a standard, valid and reliable way?
5. Was exposure measured in the same way for cases and controls?
6. Were confounding factors identified?
7. Were strategies to deal with confounding factors stated?
8. Were outcomes assessed in a standard, valid and reliable way for cases and controls?
9. Was the exposure period of interest long enough to be meaningful?
10. Was appropriate statistical analysis used?
